# Alternative Transcription Start Site Usage and Functional Implications in Pathogenic Fungi

**DOI:** 10.3390/jof8101044

**Published:** 2022-10-03

**Authors:** Thi Tuong Vi Dang, Jessie Colin, Guilhem Janbon

**Affiliations:** 1 Unité Biologie des ARN des Pathogènes Fongiques, Département de Mycologie, Institut Pasteur, Université de Paris Cité, F-75015 Paris, France; 2 Ecole Pratique des Hautes Etudes, PSL Research University, F-75014 Paris, France

**Keywords:** alternative transcription start sites, fungi, RNA

## Abstract

Pathogenic fungi require delicate gene regulation mechanisms to adapt to diverse living environments and escape host immune systems. Recent advances in sequencing technology have exposed the complexity of the fungal genome, thus allowing the gradual disentanglement of multiple layers of gene expression control. Alternative transcription start site (aTSS) usage, previously reported to be prominent in mammals and to play important roles in physiopathology, is also present in fungi to fine-tune gene expression. Depending on the alteration in their sequences, RNA isoforms arising from aTSSs acquire different characteristics that significantly alter their stability and translational capacity as well as the properties and biologic functions of the resulting proteins. Disrupted control of aTSS usage has been reported to severely impair growth, virulence, and the infectious capacity of pathogenic fungi. Here, we discuss principle concepts, mechanisms, and the functional implication of aTSS usage in fungi.

## 1. Introduction

RNA molecules are synthesized in the cell by DNA- or RNA-dependent RNA polymerases and mature through various processes, including splicing, capping, polyadenylation, methylation, editing, and endonuclease and/or exonuclease digestion [1]. The nucleotide sequence of RNA molecules and the region surrounding the corresponding gene locus play critical roles in the control of these processes and significantly influence the transcriptome structure. Typically, eukaryotic RNA polymerase promoters consist of a core promoter and associated regulatory regions [2]. The core promoter can be defined as a genomic region harboring specific sequences that allow the recruitment and assembly of the pre-initiation complex (PIC) and that prime transcription at a basal level or upon stimulating signals [3,4]. Regulatory inputs from cis-acting elements are transduced at the core promoter to regulate RNA synthesis. After the formation of the PIC, the double-stranded DNA is melted to create the “transcription bubble” at the core promoter, allowing 5′-to-3′ scanning of RNA polymerase II and transcription initiation at the transcription start site (TSS) [4]. One conserved characteristic is that transcription of a given RNA generally does not start at a unique nucleotide position. Rather, TSSs appear to form clusters in which TSS positions are located near one another. This represents the major TSS (mTSS) position, which is the most frequently used location for the initiation of RNA synthesis [4]. Each TSS cluster has a corresponding core promoter that spans the cluster and primes transcription initiation [4]. While core promoters are characterized by elements such as the “TATA box”, initiator element (Inr), and/or TFIIB recognition element (BRE), gene-specific regulatory elements are located upstream of the core promoter and control transcription [2,3,4,5].

The general transcription machinery is highly conserved across eukaryotes. Accordingly, fungi share similarities with mammals, including cofactors, cis-elements that control RNA pol II initiation, and other parts of the transcription process [2,3,6,7,8]. In *Saccharomyces cerevisiae*, the TATA box is typically located at positions from 40 to 120 bp upstream of the TSS [3,9]. This distance between the TATA box and the TSS seems to be organism dependent. For example, this distance is shorter in *Schizosaccharomyces pombe* (40 to 70 bp) [10,11,12,13]. Core promoters of genes in many pathogenic fungi, such as *Ustilago maydis crg1* and *mig2-1* to *mig2-6*, *Nectria haematococca PDA1*, and *Coprinopsis cinerea clp1*, contain a TATA box located around 40 to 46 bp upstream of TSS [14,15,16]. Likewise, BRE is found in the *Magnaporthe grisea MPG1* gene, while analysis of 19 oomycete *Phytophthora infestans* genes revealed an Inr sequence that is highly similar to that of mammals [14,17,18]. In human fungal pathogens, a genome-wide analysis in *Candida glabrata* showed that the TATA box exists within the region from 200 to 1 bp upstream of the TSS [19]. In *Cryptococcus neoformans* and *Cryptococcus deneoformans*, the TATA box, when present, can be found 35–40 bp upstream of the mTSS (Dang and colleagues, manuscript in preparation).

Recently, the analysis of the distance between the TATA box and TSS in 12 yeast species allowed the definition of two classes of transcription initiation mechanisms [20]. The first mechanism, referred to as the “classic model” because it seems to be more widespread among eukaryotes, involves direct recognition of the TSS from factors recruited to the PIC. The other model, referred to as the scanning model, is based on the recruitment of RNAPII to the PIC, the opening of the transcription bubble, and scanning by RNAPII from the PIC downstream region towards the 3′ end to find an acceptable TSS. *Candida albicans* is more likely to use the scanning model, while *Cryptococcus* utilizes the classic model of transcription initiation.

The historical model of gene expression is that a gene is transcribed into a single mRNA that eventually serves as a template for the synthesis of a functional protein. The Beadle and Tatum “one gene, one enzyme” postulate imprinted the minds of scientists for decades before diverse transcriptional and post-transcriptional diversity was described. The textbook definition of a gene is often given as a transcription unit with one core promoter; thus, a gene would have only one TSS. However, recent advances in sequencing technology have allowed a closer look at the diversity of RNA molecules, revealing the spectacular plasticity of the eukaryotic transcriptome [21,22,23,24]. In mammals, usage of alternative splicing (aSpl),) alternative TSSs (aTSSs), and alternative Poly(A) sites (aPAS) is prominent [25,26,27,28,29]. These processes are developmentally regulated [30], and defects in these processes are associated with a number of genetic diseases, such as thalassemia, retinitis pigmentosa, heart failure, and various types of cancer [31,32,33,34,35,36,37,38]. Interestingly, quantitative analysis of TSS usage suggested that aTSS and aPAS usage represents a major source of transcript isoform diversity in human tissues [39]. Thus, aTSS usage can have consequences on RNA stability, localization, and coding potential. aSpl, aTSS, and aPAS appear to be common in fungi [40]. However, these processes mostly result in unproductive transcripts, which have a limited impact on proteome diversity and thus the processes function more as a way to regulate gene expression [40,41,42,43,44]. Due to technical difficulties associated with their study, knowledge on aTSS was, up to recently, restricted to the model yeasts *S. cerevisiae* and *S. pombe*. Although less is known about pathogenic fungi, existing data suggest that these mechanisms might contribute to fine-tuning gene expression during infection [45,46,47]. Here, we review recent literature on aTSS usage and regulation and the consequences on fungal biology. We focus on fungal pathogens and discuss the potential and the mostly unexplored consequences of these regulations on virulence.

## 2. Identification of Alternative Transcription Start Sites

An aTSS is a TSS cluster that differs from the reference TSS most frequently used to initiate RNA synthesis under various environmental conditions. In the scanning model, the PIC may assemble at a single location but initiates transcription at different aTSS clusters [48,49]. For clarity, we will consider aTSS to have a similar meaning to alternative TSS cluster, alternative promoter, or promoter switch, and the term is not related to the heterogeneity of TSSs within a TSS cluster as mentioned above. More than half of human genes have alternative promoters, and a human gene has an average of 4 TSSs [50]. This implies that a single human gene can harbor multiple core promoters that drive the transcription of RNA polymerase at distinct TSSs. Recently developed techniques, such as CAGE, TIF-seq, TL-seq, TSS-seq, and STRIPE-seq, provide insight into the TSS position at a single-nucleotide resolution and have confirmed the model of multiple TSS genes in eukaryotes [51,52,53,54,55]. Kimura and colleagues reported that at least 7674 genes, accounting for 52% of total human annotated genes, were regulated by putative alternative promoters [56]. In *Drosophila*, data from 5′ cap read sequencing revealed 34,664 discrete TSS clusters associated with 8577 genes, suggesting comparable complexity of aTSS usage in the fruit fly compared to humans [57]. The situation appears to be similar in fungi. For instance, 1773 *S. pombe* genes have at least two core promoters [58]. In *S. cerevisiae*, 56% of the genes have at least two TSS clusters, and alternative core promoter usage by a gene is widespread in response to changing environments [59]. Nevertheless, information about aTSS usage in pathogenic fungi is limited due to both the scarcity of genome-wide studies and caveats in data integration. Results from 5′-end-cDNA sequencing of *C. glabrata* showed that out of 4316 coding genes, only 10% (n = 435) were transcribed from a single TSS, but it is unclear as to how many TSS clusters these TSSs belong [19]. A study in *Aspergillus nidulans* grouped 18,817,969 TSS positions into 17,992 putative TSS clusters, but further analysis of alternative usage of these clusters in various growth conditions has yet to be conducted [60]. Nonetheless, evidence of aTSS usage in pathogenic and non-pathogenic fungi has been published. In *Neurospora crassa*, the TSS positions of the circadian clock gene frq are distributed into two alternative major clusters depending on light exposure of the fungus [61]. In *C. neoformans*, aTSS usage results in the production of two alternative isoforms of *PUM1* regulated by the sexual development growth phase [62]. Our recent TSS-seq data analysis in two *Cryptococcus* species revealed the existence of more TSS clusters than coding genes, suggesting widespread aTSS usage in these pathogenic yeasts [63]. While global studies will provide more understanding of the extent and dynamics of aTSS usage in various fungal species, the biological functions and mechanism of aTSS in pathogenic fungi are of particular interest.

## 3. Biological Consequences of aTSS in Fungi

During the 2000s, promoter switching events associated with mammalian development and disease etiology have been the subject of reviews that have cited results from hundreds of studies [64,65,66,67]. aTSS usage is expected to have a similar impact on the fungal transcriptome and proteome. Although aTSS likely affects lncRNAs in addition to mRNAs, we focus on mRNAs and discuss the consequences of aTSS usage on fungal mRNA level, mRNA diversity, and proteome diversity.

### 3.1. Alternative Transcript Isoforms Share the Same ORF but Differ in Leader (5′UTR) Sequence

**Figure 1:**
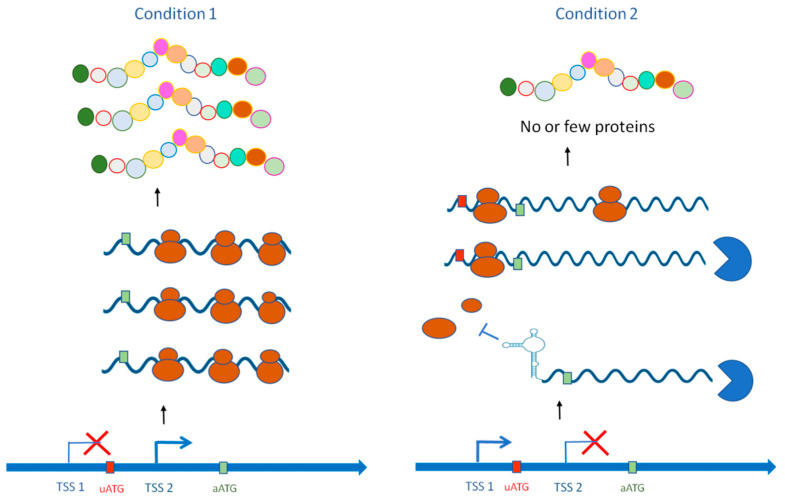
aTSS usage regulates gene expression without impacting protein diversity. aTSS usage includes or excludes regulatory elements within the transcript leader sequence, such as the uORF or secondary structure. This regulates the translation, stability, or subcellular targeting of the produced mRNA.

aTSSs can be found upstream or downstream of the annotated TSS, which modulates the 5′UTR length. This modulation can result in the inclusion or exclusion of sequences containing important regulatory elements (Figure 1). For instance, the presence of one or more upstream open-reading frames (uORFs) within this region can dramatically alter mRNA stability and translation efficiency. In mammals, an uORF-linked regulatory mechanism has been illustrated in the mouse with the cyclin-dependent kinase inhibitor (p18INK4c) and peroxisome proliferator-activated receptor (PPAR) beta/delta [68,69]. In *S. cerevisiae*, 791 mRNAs contain uORFs in their transcript leader sequence [70], and 252 genes contain conserved uORFs [71]. Although the function of most uORFs has not been explored in detail, examples of uORF-dependent regulation associated with alternative TSS usage exist. For instance, alternative promoter usage of the *NDC80* gene produces either a nonfunctional long transcript bearing several uORFs or a short translatable mRNA, allowing versatile management of protein expression [72]. The *NDC80* gene encodes a subunit of the outer kinetochore, which plays a key role in meiosis. The controlled expression of two *NDC80* mRNA isoforms is responsible for the inactivation and reactivation of the kinetochore during cell division through the Ndc80 protein level. The translatable short isoform is highly expressed in vegetative growth, but at the beginning of meiosis, 100% of cells express only the nonfunctional long isoform, which harbors nine uORFs. Hence, no Ndc80 protein is produced at the meiotic prophase, thereby ensuring proper removal of the kinetochore at this stage. Mutating all nine AUGs led to an increase in Ndc80 protein during prophase, confirming the inhibitory effect of uORFs on the translation of the main coding sequence. This abnormal expression of *NDC80* from the mutated long mRNA isoform led to abnormal chromosome segregation during cell division. In pathogenic fungi, uORFs in the 5′UTR contribute to the control of translation and RNA stability in response to environmental cues [73,74]. In *C. neoformans*, a robust, transient transcriptome modification is triggered upon exposure to reactive oxygen species [75], which is explained by translation inhibition through Gcn2-mediated eIF2α phosphorylation [73]. This likely favors the efficient translation of the oxidative stress response genes *ERG110* and *GCN4*, which both contain uORFs in the transcript leader (TL) of their mRNA [73,76]. In addition, the production of an alternative transcript isoform containing an uORF has been reported for the *Metarhizium robertsii* Mr-OPY2 gene [43]. The plant- and fly-infecting fungus *M. robertsii* requires precise regulation of the Mr-OPY2 membrane anchor protein during saprophyte-to-insect pathogen transition. During saprophytic growth, Mr-OPY2 mRNA contains two uORFs that impede the translation of the main coding sequence. The elevated level of Mr-OPY2 protein during infection stages is achieved through the production of a short mRNA isoform that does not contain the two uORFs. Mutant strains that fail to express Mr-OPY2 proteins were impaired in appressorial formation and infection capacity. Artificial constitutive expression of the major open reading frame (ORF) during saprophytic growth results in the fluffy phenotype, aberrant conidiophores, and significantly reduced conidial production. These findings illustrate that aTSS- and uORF-associated regulatory strategies are exploited by both fungi and mammals as a flexible tool to regulate protein levels.

Genome-wide data analyses of TL structure performed in *Saccharomyces*, *Cryptococcus, Aspergillus*, *Candida*, and *Neurospora* species confirmed the repressive effect of uORFs on translation efficiency [53,60,63,77]. The translation efficiency of annotated ORFs is negatively correlated with the number of uORFs present in the 5′UTR. This is in agreement with the observation in mouse fibroblasts that uORF-containing longer mRNA isoforms are enriched in the monosome fraction, which is indicative of a lack of translation of the main ORF [78]. Analysis of Ribo-seq data revealed some examples of such regulation in *Cryptococcus*. At the CNAG_06246 and CNAG_03140 loci, only uORF translation was observed in the tested conditions, whereas the main ORF translation was completely abolished, suggesting tight regulation of gene expression [63].

Although the general view is that uORF translation would eventually trigger mRNA to degrade via nonsense-mediated mRNA decay (NMD), as suggested in *Cryptococcus* and *S. cerevisiae* [53,63], some uORF-based regulation appears to be more complex [79]. A classic example is the *S. cerevisiae GCN4* gene, which has four small uORFs in the 5′ leader sequence of its mRNA [80]. In amino acid-rich media, ribosomes are dissociated after passing a GC-rich region at the end of the fourth uORF within *GCN4* mRNA. Therefore, ribosome re-initiation scanning is hindered after translation of the fourth uORF, and no protein is synthesized from the main ORF.

Given the small length of uORF-born peptides, one question is whether these short peptides possess any biological function. Examples of functional small peptides (<100 amino acids in length) are available in vertebrates and *Drosophila* [81]. In fungi, *N. crassa* arg-2 mRNA contains an evolutionarily conserved uORF coding a 24-amino acid peptide called arginine attenuator peptide (AAP), which participates in the control of ribosome movement [82]. Nevertheless, the features of repressive uORFs are evolutionarily maintained rather than their amino acid sequence [83]. This suggests that conserved uORFs are mostly involved in the regulation of translation of the downstream major ORF rather than encoding functional protein [84].

A longer 5′ leader sequence of transcripts resulting from an upstream aTSS can potentially form a secondary structure that interferes with translation initiation [85]. Once assembled, the 43S complex scans the TL sequence to identify the correct translation start site [86]. Though the ribosome has the ability to process helicase activity, RNA secondary structures, such as the stem-loop hairpin structure, can block translation initiation via blockage of the scanning process [87,88]. For instance, under hypoxic conditions, *S. pombe* transcribes a translationally silent transcript from an upstream promoter at the *TCO1* locus [89]. The additional 751-nucleotide sequence of this alternative transcript is computationally predicted to form a stable stem-loop structure thought to block translation initiation by impairing 43S scanning.

Additional cis-regulatory sequences included within the 5′ leader sequence upon aTSS usage can alter mRNA translation potential. An interesting example is the Pumilio protein family *PUM1* gene in *C. neoformans* [62]. Pum1 is an RNA-binding protein required for hyphal formation and is known to indirectly enhance the mRNA stability of the master filamentation regulator Znf2 [90]. In yeast cells, the Pum1 protein binds to its own transcript leader sequence, blocking ribosome scanning and shutting off mRNA translation [62]. In filaments, *C. neoformans* utilizes an alternative downstream TSS to produce a shorter isoform lacking the Pum1 binding site [62]. Thus, aTSS usage prevents auto-inhibition, thereby allowing full expression of *PUM1* and activation of *ZNF2*.

### 3.2. Alternative Transcripts Are Translated into N-Termini-Truncated Proteins

**Figure 2:**
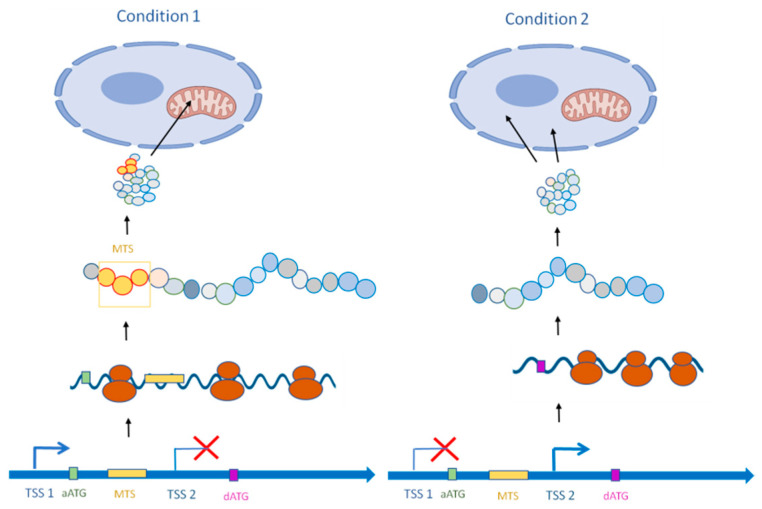
Alternative transcripts leading to N-terminus-truncated proteins with different localization to that of the canonical protein. Condition 1: mRNA is transcribed from TSS1. This mRNA is translated into a protein with a localization target signal (mitochondria targeting signal [MTS] in this example) at its N terminus. The protein is targeted to the mitochondria. Condition 2: mRNA is transcribed from TSS2. The shorter transcript isoform is translated into an N-terminus-truncated protein lacking the MTS. The resulting alternative protein localizes to cytosol.

Although aTSS usage maintains an intact ORF in 60% to 80% of studied cases in mammals, it can also be a source of proteome diversity [65]. Thus, the usage of TSS clusters within coding sequences potentially results in novel protein isoforms. The translationally active downstream ATG (dATG) is typically found in-frame with the annotated ATG (aATG), so the alternative protein is an N-terminus-truncated version of the annotated one. This shorter polypeptide can maintain the same functional domains but lacks sequences critical for protein localization (Figure 2). For instance, the plant *Arabidopsis thaliana* growing in shaded conditions uses aTSS to produce a cytosolic isoform of *GLYK*, a photorespiration enzyme believed to solely reside in the chloroplast [91]. Such aTSS regulation of protein localization by N-terminus truncation has been described for some *S. cerevisiae* tRNA synthetase genes. In these examples, instead of having a dedicated gene for cytosolic and mitochondrial tRNA-synthetase, a single gene generates both enzymes [92]. Both cytoplasmic and mitochondrial versions of histidinyl, valinyl, and cysteinyl tRNA synthetases are encoded by a single gene (*HST1*, *VAS1,* and *CRS1*, respectively) [92,93,94]. At these loci, aTSSs generate either a long or short transcript isoform leading to alternative protein versions of Hts1, Vas1, and Crs1. The truncated versions lack the mitochondria targeting signal (MTS) at the N terminus and remain in the cytosol, while the full-length versions are translocated into the mitochondria. In the fungal pathogen *C. neoformans*, both tRNA synthetase activities are encoded by a single gene for most amino acids [63]. The expression of the two protein isoforms can be controlled by aTSS usage to produce a long and a short transcript, and the long isoform can produce both cytosolic and mitochondrial enzymes. Another layer of regulation is the selection of the translation initiation position, which is based on the consensus level of the associated Kozac context of the long transcript [63]. However, aTSS-dependent protein localization has been reported for other *C. neoformans* genes. For instance, *C. neoformans* synthesizes a long *UVE1* mRNA isoform which codes a protein, following UV exposure, specifically targeted to the mitochondria [95]. This long Uve1 protein is functionally active as a DNA damage repair endonuclease and protects the mitochondrial genome from potentially lethal UV-induced DNA damage. Similarly, *C. neoformans* employs aTSSs as a novel layer of regulation of superoxide dismutase activities. The genes *SOD1* and *SOD2* encode genes with cytoplasmic Cu-dependent and mitochondrial manganese-dependent superoxide dismutase activities, respectively [96]. Upon Cu shortage, an aTSS is used to regulate both gene products: a translationally repressed *SOD1* mRNA is produced while a shorter version of *SOD2* mRNA produces a functional cytosolic version of manganese-dependent superoxide dismutase Sod2. Thus, during infection, a condition in which copper is limited in host cells, the *Cryptococcus* manganese-dependent superoxide dismutase typically located in the mitochondria is translocated to the cytosol to maintain the cytosolic redox equilibrium against oxidative stress [96]. TSS switch-driven subcellular localization of proteins is not restricted to mitochondrial and cytosolic targeting. For instance, aTSS usage regulates the alternative production of the secreted, glycosylated version of the *S. cerevisiae* invertase *SUC2* in glucose-rich culture instead of its constitutive intracellular, non-glycosylated form [97].

**Figure 3:**
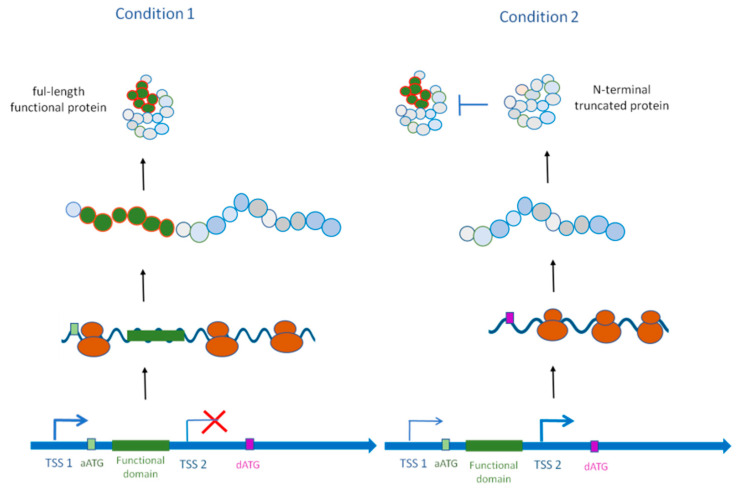
Alternative TSS usage leads to the production of an N-terminal-truncated protein with an alternative function. Condition 1: The mRNA is transcribed from the TSS1. The produced protein contains the functional domain (green). Condition 2: The mRNA is transcribed from the TSS2. The aTSS (TSS2) is located within the annotated coding sequence. The resulting protein does not contain the functional domain and might function as an inhibitor of the full-length protein, as in the case of the *Fusarium graminearum* protein Zeb2.

Alternative transcription initiation can be used to produce a shorter protein that loses important functional domains (apart from the localization signal) and in turn acts as an inhibitor of its full-length counterpart (Figure 3). An example in fungi is the *ZEB2* gene of the cereal-infecting fungus *Fusarium graminearum* in which aTSS not only impacts protein localization but also alters its function [98]. The full-length Zeb2 protein (Zeb2L) localizes exclusively to the nucleus. This long isoform has a basic leucine zipper (bZIP) DNA-binding domain and functions as a transcription factor that induces the production of the polyketide mycotoxin zearalenone (ZEA), a chemical compound leading to hyperestrogenic syndrome in infected cereals. ZEA accumulation triggers the synthesis of an N-terminally truncated protein (Zeb2S) that lacks the bZIP domain and exists in both the nucleus and cytoplasm. The short isoform forms heterodimers with Zeb2L and thus impedes the DNA-binding activity of Zeb2L in an autoregulatory process [98]. Similarly, in *S. pombe*, wtf genes encode killer meiotic drivers which are selfish DNA sequences. Interestingly, the wtf drivers use alternative TSS to produce two protein isoforms [99]. Here, the long protein isoform Wtf4^antidote^ neutralizes the short protein isoform Wtf4^poison^ via heterodimerization. Moreover, the expression of both isoforms is tightly controlled in timing and localization to give the driver a transmission advantage into the next generation [99].

aTSS can result in the production of alternative protein isoforms encoded by alternative ORFs. To our knowledge, there are only two characterized examples in mammals: cyclin-dependent kinase inhibitor 2A gene (CDKN2A) and p21 in humans [100,101]. Each encodes two protein isoforms with different reading frames resulting from an alternative promoter coupled with different splicing patterns. Until recently, reports of similar aTSS-induced out-of-frame proteins have been unavailable in fungi. However, genome-wide proteomics approaches that sequence the N-terminal peptide have enabled studies on the impact on the proteome and the discovery of out-of-frame peptides [102,103,104]. These putative detected out-of-frame peptides are clearly not degradation products of annotated proteins that have a different reading frame. Rather, they likely result from bona fide proteins that are translated starting from an out-of-frame dATG or an in-frame dATG in the presence of alternative splicing. It is likely that such alternative proteins exist in pathogenic fungi, but their impact on the biology and virulence of these organisms remains to be studied.

### 3.3. Transcript Isoforms Are Similar in Coding Sequence and Translational Efficiency

**Figure 4:**
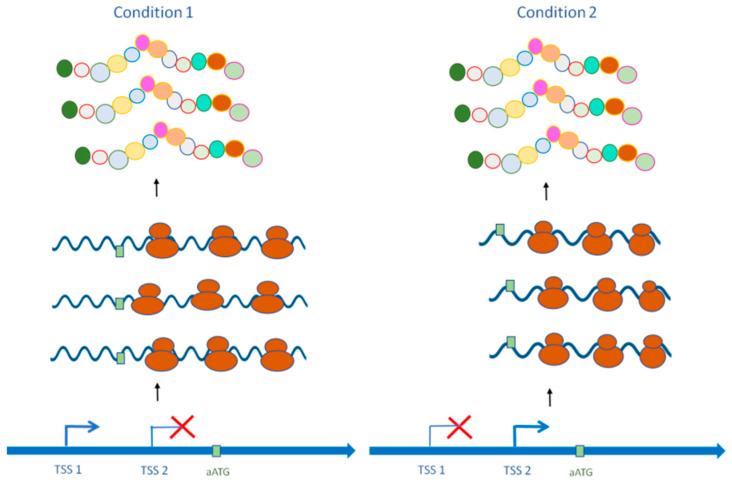
Alternative transcript isoforms code identical proteins and possess the same translation capacity. Condition 1: Canonical mRNA from TSS1 is produced. Condition 2: Alternative mRNA from TSS2 is transcribed. The alternative TSS results in transcript isoforms that differ in the 5′ UTR but have the same coding sequence. This alternative isoform does not significantly impact stability or translational efficiency.

In some cases, aTSS usage does not impact protein output or translation efficiency (Figure 4). Possible explanations of multiple promoters for essential genes include ensuring expression level regardless of diverse initiation environments, such as the available transcription factor pool, or diminishing the fatal effects of mutations within the promoter [66]. For example, in the fungus *Aspergillus oryzae*, most glycolysis-reversible enzyme-coding genes have multiple TSSs even though they are constitutively expressed [105]. Carbon source-dependent aTSS usage is only observed in two genes: those encoding enolase (enoA) and fructose-bisphosphate aldolase (fbaA), where two promoters are alternatively used in response to nutritional signals. Thus, with the exceptions of enoA and fbaA, the existence of multiple TSSs in other glycolysis/gluconeogenesis genes probably functions to maintain a constant level of protein in any environmental condition. On the other hand, enoA is an interesting case because the use of the two promoters depends on the carbon source. This gene is transcribed into two mRNAs that differ only in the 5′UTR; the short isoform is specific to glycolytic conditions, and the long transcript is highly expressed in gluconeogenic conditions. Replacing the 5′UTR of one enoA mRNA isoform with another 5′ UTR of the reporter gene does not alter translational activity. However, mutations abolishing transcription from the upstream TSS prevent cell growth in acetate, while transcription from the downstream TSS is important for cell proliferation in glucose. Thus, *A. oryzae* requires transcription of enoA from distinct TSS/promoters for environmental adaptation, but no clear functional differences between the two mRNA isoforms can be observed. Here, *A. oryzae* might use aTSSs to adapt to the different availability of some transcription factors so as to satisfy the different demands of an important reversible enzyme in glycolysis and gluconeogenesis.

## 4. Mechanism of Alternative TSS Usage Control

As discussed in the examples above, some transcripts produced from alternative TSSs are functional and critical to the cell. Fungi actively control their synthesis by employing both cis-elements and trans-factors to accurately produce the correct mRNA isoforms at the correct time.

### 4.1. Transcription Factors Regulate Alternative TSS Usage

Transcription can be initiated from aTSS via binding of transcription factors (TFs) to its dedicated promoter, with evidence available both in mammals and yeasts. The promoter switch of the rodent gene satb1 during T-cell development is controlled by the transcription factor TCF1 [85]. In *S. pombe*, transcription from the upstream aTSS of the tco1+ gene in hypoxic conditions is triggered by the oxygen-sensitive TF Sre1 [89]. *S. cerevisiae* Gcn4 is a striking example of a TF that induces unconventional transcription: 546 Gcn4 ChIP-seq peaks were enriched during amino acid starvation vs control nutrient-rich media. Nearly 60% of Gcn4 genic binding sites are not located at the canonical promoter region, but rather are found inside coding sequences (CDSs) [106]. Many of these Gcn4 signals within CDSs are adjacent to induced TATA-binding protein peaks, suggesting the existence of cryptic internal promoters. Indeed, Gcn4 binds to its site within the ORF of *POS1*, *SNX41*, *SPO21*, and *COG1* and induces both the noncanonical antisense and sense transcriptions starting around 100 bp upstream or downstream. The H3 histone occupancy profile suggests that Gcn4 does not bind to the pre-existing nucleosome-depleted region. Rather, Gcn4 binding is more likely to stimulate gentle histone removal at the surrounding region, suggesting that Gcn4 actively provokes transcription initiation. This implies active regulatory activities of Gcn4 on aTSS usage.

TFs function not only as inducers but also as repressors of alternative promoters. Examples in mammals include the transcription factor Chx10, which negatively regulates the synthesis of the two transcript isoforms (H- and D-Mitf), but not eight other isoforms of the MIFT gene, in the retina during murine eye development [107]. Examples of TFs that negatively control the transcription of a particular alternative mRNA isoform have yet to be discovered in yeast. However, *S. cerevisiae* transcription factor Rap1 is reported to bind at the promoters of highly expressed genes and repress pervasive transcription from these promoters, possibly by contributing to nucleosome positioning [108,109].

TFs are reported to mediate feedback regulation on aTSS usage at their encoding gene. In humans, the pluripotent stem cell transcription factor NANOG binds to and auto-upregulates the usage of its own proximal promoter [110]. In *C. neoformans*, the RNA-binding protein Pum1 autoregulates the expression of its encoding gene by binding *ZNF2* mRNA, thus regulating the expression of this TF [62]. In turn, Znf2p binds to the *PUM1* proximal promoter, inducing the synthesis of a short isoform that lacks the sequence required for translational repression as in the long isoform [62].

Identification of the TFs regulating aTSS usage is suggested by the analysis of the sequence surrounding these TSSs and confirmed through genetic and/or ChIP-based analyses [85,111,112]. For instance, the binding site of 2 TFs in *A. oryzae* (AcuK and AcuM) are detected at the upstream promoter of the enoA gene, suggesting that these TFs could regulate the expression of the long transcript of the enoA gene in glucose starvation conditions. Accordingly, mutating the potential binding motif of AcuK and AcuM at the upstream promoter of the enoA gene reduces the expression of the long transcript in acetate culture conditions but not in glucose-rich conditions [105].

Some TFs can affect aTSS usage of multiple genes, as observed in mammals and plants [91,113]. In *S. cerevisiae*, the TFs Ume6 and Ime1 form a heterodimer to activate transcription from alternative promoters upstream of meiotic genes specifically during the meiotic prophase. This allows the downregulation of these meiotic-specific genes through the combined action of translational and transcriptional repression. Thus, the production of 5′ extended transcripts down-regulates the expression of the short isoforms through transcriptional interference of the downstream promoters. Whereas, these long mRNA isoforms are enriched in uORFs, leading to translational repression of the main ORF [114]. Accordingly, Ume6 binding sites are highly enriched at the promoter of these non-canonical transcripts. Similarly, Gcn4 likely functions as the master regulator controlling the use of a number of aTSSs at different loci in response to amino acid limitation [106]. In addition, Zap1 regulates aTSS usage at the *RTC4* and *RAD27* loci through zinc-responsive elements (ZREs) [115]. In *C. neoformans*, the TF Cuf1 controls aTSS usage at the *SOD1* and *SOD2* loci via Cu-responsive elements (CuREs) [96].

### 4.2. Cis Regulatory Transcription Activity and Chromatin Conformation Remodeling Control Alternative Transcription

**Figure 5:**
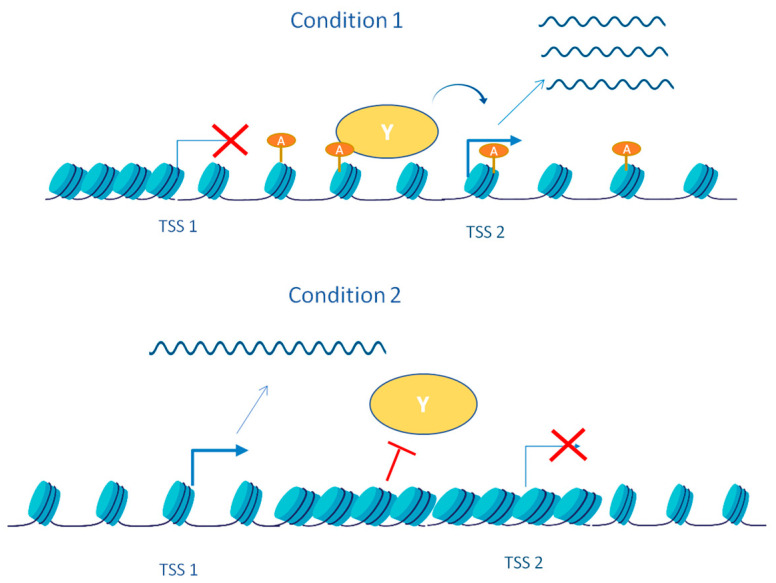
Regulatory action in cis and chromatin conformation control alternative transcription. Condition 1: There is no transcription from TSS1. Acetylated histones (orange circle) at the gene body downstream TSS1 are associated with a relaxed chromatin state. The transcription factor Y, which activates transcription at the TSS2, can bind. Condition 2: Transcription from TSS1 leads to histone deacetylation. This reduction in histone acetylation leads to a more condensed chromatin conformation that occludes the binding of transcription factor Y, thereby repressing transcription from TSS2.

Either alternative promoters are independently activated by different pathways via distinct transcription factors, or their usages are intertwined with each other through transcriptional interference. In *S. cerevisiae*, transcription through a promoter can impede its activity [72,116] (Figure 5). For instance, transcription of the short functional *NDC80* mRNA isoform is inhibited by the transcription event starting upstream that generates a 5′ extended isoform [72]. This repression likely results from co-transcriptional chromatin remodeling [117,118]. During transcription from the upstream TSS of *NDC80*, the histone methyl transferases Set1 and Set2 regulate H3 lysine 4 dimethylation (H3K4me2) and H3 lysine 36 trimethylation (H3K36me3) levels, respectively [119]. Global mapping of epigenetic imprinting showed that H3K36me3 and, to a lesser extent, H3K4me2 are strong predictors of transcription repression mediated by upstream transcription [114]. The repressive histone marks H3K4me2 and H3K36me3 are read by histone deacetylases Set3 and Rpd3S, respectively, to promote histone deacetylation at these regions [119]. Histone deacetylation enhances the electrostatic interaction between histones and DNA, thus promoting nucleosome occupancy and inhibiting local transcription initiation [120]. Transcription activity from upstream TSSs is likely associated with low-level histone acetylation followed by a tighter wrapping of the histone core by the DNA string at the gene body. The converse can be true, where limited upstream transcription activity brings about permissive histone marks at the gene body, thereby allowing downstream transcription. For instance, low transcriptional activity of *S. cerevisiae* lncRNA *IRT2* induces the acetylation of histone H3 lysine 56 (H3K56ac). The associated relaxed chromatin conformation allows binding of the TF Rme1 at the promoter of lncRNA *IRT1*, which is located downstream and facilitates its transcription [121]. However, not every gene expressing 5′ extended transcripts is associated with a reduction in the abundance of the short transcripts [114]. Further, the effect of expressing distal promoters on proximal ones can range from repression to activation [122]. Interestingly, this effect appears to be dependent on the distance between the distal and proximal TSS. Enhanced transcription from the upstream TSS can repress transcription from the downstream TSS if located more than 80 bp from one another, while a smaller distance is linked to a positive correlation of their usage [122]. It is possible that a downstream TSS distantly separated from the upstream TSS is likely to have its own promoter, so its activity is regulated by transcriptional interference. Nevertheless, this cis-regulation through transcriptional interference of aTSS usage remains to be described in pathogenic fungi.

In many situations discussed above, it is puzzling that the cell uses so many resources on synthesizing a translationally deficient mRNA. A possible explanation for this “unnecessary” energy consumption resides on the regulatory cis action of alternative transcription as discussed above. In *S. cerevisiae*, aTSS usage driven by alternative promoters has cis-regulatory activities, mostly via transcriptional activation. However, even if the RNA species produced from these aTSSs are not coding/translated, we cannot exclude that at least some may have additional trans-regulatory activities. In metazoans, several examples exist of trans-acting lncRNA acting on chromatin through binding chromatin modifiers or remodelers [123]. Thus, it is possible that this regulatory activity could occur in fungi as well.

Though not yet reported in yeast and fungi, several lines of evidence suggest the involvement of DNA methylation in the regulation of aTSS usage in mammals. Cap-targeted and bisulfite genomic sequencing data reveal variation in methylation patterns among alternative promoters of tissue-biased genes [124]. In humans and viruses, the activity of alternative promoters is negatively correlated with hypermethylation of its CpG islands [124,125,126,127,128]. In conditions in which demethylation of aTSS is observed, the active promoter-associated histone modification H3K4me3 is detected spanning the promoter, implying genuine transcriptional activities [124,125]. How DNA methylation and TFs interfere with each other, and impact promoter expression is still controversial. In the human gene garp, an in vitro DNA-protein binding assay demonstrated an inhibitory effect of methylated CpG flanking the alternative promoter on the binding capacity of the 2 positive transcriptional regulators NFAT and Foxp3 [124]. Thus, steric hindrance of the methyl groups associated with DNA methylation at alternative promoters can repress TF binding to the corresponding cis-elements [129]. Conversely, TF binding can also affect local DNA methylation patterns and induce transcription through passive demethylation of the bound region [129,130]. Although cytosine methylation has been identified in at least 16 yeast species [131], the impact of DNA methylation on transcription requires clarification in model yeasts and pathogenic fungi.

## 5. Conclusions and Remaining Challenges

aTSS usage is a major mechanism regulating gene expression and proteome diversity in eukaryotes. Use of alternative promoters results in alterations in mRNA that can significantly modify transcript stability and translational efficiency, as well as protein sequence localization and function. Thus, aTSS has a potential critical impact on cell growth, differentiation, and adaptation. This type of regulation of both the transcriptome and the proteome structure is still poorly studied in pathogenic fungi. However, data produced in fungal model organisms, such as *N. crassa*, *S. cerevisiae*, and *S. pombe*, suggest that regulation of gene expression via aTSS may be common in pathogenic fungi as well. Accordingly, genome-wide analysis in two species of *Cryptococcus* and in *C. albicans* identified several aTSS clusters associated with coding genes, although the regulation of their expression and the associated molecular mechanisms remain to be explored [20,63]. Fungal pathogens need to adapt to diverse conditions and efficiently produce virulence factors to escape the host immune system and potentially acquire drug resistance. This suggests sophisticated and precise gene regulation mechanisms, such as aTSS usage, to regulate their biology and virulence.

The analysis of aTSS usage in model organisms revealed a significant number of multiple-promoter genes showing no promoter switch in different conditions. In this case, it is possible that transcript isoforms are regulated under unknown conditions. They can also be co-regulated. Cells uniformly express both alternative transcripts across conditions, but a population of cells could express a specific isoform while the alternative transcript would be expressed in the other part of the cell population. In the latter scenario, analysis of the whole population at once does not capture the true picture of aTSS usage [132]. Thus, single-cell TSS analysis would allow more accurate findings. However, this type of analysis is challenging in fungi. TSS likely plays a major role in host–pathogen interactions given the heterogeneity of in vivo and in vitro fungal populations [133].

Overall, the handful of studies on aTSSs in pathogenic fungi suggest wide usage and should prompt exploration of the mechanisms regulating the expression of virulence factors, drug resistance, or in vivo fungal cell biology. Although a limited number of sequence datasets have been produced, no genome-wide analysis of the regulation of aTSS usage has been performed in any pathogenic fungi.

## Data Availability

Not applicable.
